# Effect of *BRAF* mutational status on expression profiles in conventional papillary thyroid carcinomas

**DOI:** 10.1186/1471-2164-16-S1-S6

**Published:** 2015-01-15

**Authors:** Hans-Juergen Schulten, Reem Alotibi, Alaa Al-Ahmadi, Manar Ata, Sajjad Karim, Etimad Huwait, Mamdooh Gari, Khalid Al-Ghamdi, Faisal Al-Mashat, Osman Abdel Al-Hamour, Mohammad Hussain Al-Qahtani, Jaudah Al-Maghrabi

**Affiliations:** 1 Center of Excellence in Genomic Medicine Research, King Abdulaziz University, Jeddah, Saudi Arabia; 2 KACST Technology Innovation Center in Personalized Medicine, King Abdulaziz University, Jeddah, Saudi Arabia; 3 Department of Biochemistry, King Abdulaziz University, Jeddah, Saudi Arabia; 4 Department of Surgery, Faculty of Medicine, King Abdulaziz University, Jeddah, Saudi Arabia; 5 Department of Surgery, King Faisal Specialist Hospital and Research Center, Jeddah, Saudi Arabia; 6 Department of Pathology, Faculty of Medicine, King Abdulaziz University, Jeddah, Saudi Arabia; 7 Department of Pathology and Laboratory Medicine, King Faisal Specialist Hospital and Research Center, Jeddah, Saudi Arabia

**Keywords:** Oligonucleotide microarrays; expression profiling; papillary thyroid carcinoma (PTC); *BRAF* mutation; network analysis; canonical pathways

## Abstract

**Background:**

Whereas 40 % to 70 % of papillary thyroid carcinomas (PTCs) are characterized by a *BRAF* mutation (*BRAF*^mut^), unified biomarkers for the genetically heterogeneous group of *BRAF* wild type (*BRAF*^wt^) PTCs are not established yet. Using state-of-the-art technology we compared RNA expression profiles between conventional *BRAF*^wt^ and *BRAF*^mut^ PTCs.

**Methods:**

Microarrays covering 36,079 reference sequences were used to generate whole transcript expression profiles in 11 *BRAF*^wt^ PTCs including five micro PTCs, 14 *BRAF*^mut^ PTCs, and 7 normal thyroid specimens. A *p*-value with a false discovery rate (FDR) < 0.05 and a fold change > 2 were used as a threshold of significance for differential expression. Network and pathway utilities were employed to interpret significance of expression data. *BRAF* mutational status was established by direct sequencing the hotspot region of exon 15.

**Results:**

We identified 237 annotated genes that were significantly differentially expressed between *BRAF*^wt^ and *BRAF*^mut^ PTCs. Of these, 110 genes were down- and 127 were upregulated in *BRAF*^wt^ compared to *BRAF*^mut^ PTCs. A number of molecules involved in thyroid hormone metabolism including *thyroid peroxidase* (*TPO*) were differentially expressed between both groups. Among cancer-associated molecules were *ERBB3* that was downregulated and *ERBB4* that was upregulated in *BRAF*^wt^ PTCs. Two microRNAs were significantly differentially expressed of which miR492 bears predicted functions relevant to thyroid-specific molecules. The protein kinase A (PKA) and the G protein-coupled receptor pathways were identified as significantly related signaling cascades to the gene set of 237 genes. Furthermore, a network of interacting molecules was predicted on basis of the differentially expressed gene set.

**Conclusions:**

The expression study focusing on affected genes that are differentially expressed between *BRAF*^wt^ and *BRAF*^mut^ conventional PTCs identified a number of molecules which are connected in a network and affect important canonical pathways. The identified gene set adds to our understanding of the tumor biology of *BRAF*^wt^ and *BRAF*^mut^ PTCs and contains genes/biomarkers of interest.

## Background

Over the last decades the incidence rate of thyroid cancer increases worldwide [1]. In Saudi Arabia, thyroid carcinoma (TC) is considered the second most common cancer in young women [2]. About 80% of all TCs are PTCs. The majority of PTCs are histologically classified as conventional PTCs. The follicular variant of PTC (FVPTC) represents the largest subtype and accounts for about 30% of all PTCs [3]. Minor and rare subtypes include Hurthle cell variant PTC and insular PTC which bears an aggressive clinical behavior [4]. Conventional PTCs are characterized on the molecular level by a moderate to high frequency of *BRAF* mutations (40 % - 70 %) that distinguishes them from FVPTCs (10 % - 20 %) [5].

BRAF is a cytoplasmic receptor serine/threonine kinase and a key molecule in the mitogen activated protein kinase (MAPK) pathway. *BRAF* is mutated in diverse human malignancies although frequency and clinical presentation varies considerably between different types of cancers [6]. Over 90 % of all *BRAF* mutations are a valine by glycine substitution at codon 600 (V600E) in exon 15. Other *BRAF* mutations affect commonly codons adjunct to codon 600. Although the impact of *BRAF* mutations in PTC is controversially discussed, many studies found an association of *BRAF*^mut^ PTCs with unfavorable clinical features including larger tumor size, advanced tumor stage, vessel invasion, capsular invasion, tumor extension, higher risk for lymph node (LN) involvement, distant metastasis, and poor prognosis [7-10]. Consistent with this, patients with a *BRAF*^mut^ PTC are considerably older than those with a *BRAF*^wt^ PTC [5,10]. Whereas a *BRAF* mutation represents are valuable target for molecular therapy in advanced solid tumors including PTCs, molecular profiles of *BRAF*^wt^ PTCs are less known and genetic screening for valuable target genes is primarily limited to research studies [11]. The major deregulated key genes in the *BRAF*^wt^ group are *RET* and RAS. RAS consists of the highly related genes for *HRAS*, *KRAS*, and *NRAS*. Within the MAPK pathway the RAS molecules transmit signals to the downstream target *BRAF*. The most common *RET*/PTC fusions are paracentric fusions with the gene encoding *coiled-coil domain containing 6* (*CCDC6*) contributing to ~80 % and with the *nuclear receptor coactivator 4* (*NCOA4*) contributing to ~10 % of all known *RET*/PTC rearrangements. The frequency of RAS mutations and RET/PTC rearrangements differs between the populations studied and depends in part on the inclusion/exclusion criteria for the different histological PTC subtypes [12]. RAS mutations and RET rearrangements are unlikely to act as molecular drivers for onset of malignancy as they are already present in benign thyroid neoplasms [13,14]. This distinguishes them from *BRAF* mutations which are virtually absent in precursor lesions of PTCs.

Until now only a few studies compared expression profiles between *BRAF*^wt^ and *BRAF*^mut^ PTCs using array technologies [15-18]. The relevance of expressional screening in PTC according to their *BRAF* mutational status is in part related to the different clinical behavior of both PTC groups with the necessity to identify appropriate biomarkers and in part related to the different tumor biology of both groups which is not thoroughly understood [7]. In our study we took advantage of current state-of-the-art technology to screen and analyze a case series of *BRAF*^wt^ and *BRAF*^mut^ PTCs for detecting new biomarkers which could become useful to distinguish both groups on the molecular level. We did not include FVPTCs and other smaller histological subtypes of PTC in our screening to minimize expressional bias which might be related to a different histology.

## Methods

### Thyroid samples

We examined 25 specimens from PTCs and seven normal thyroid samples (TN) from patients which were treated surgically in the period between November 2008 and February 2013 at the King Abdulaziz University Hospital, Jeddah, and the King Faisal Specialist Hospital & Research Center (KFSH&RC), Jeddah. In two *BRAF*^mut^ PTCs, specimens were derived from a recurrence or a local metastasis and in one *BRAF*^wt^ PTC from an LN metastasis. Normal thyroid specimens were derived from histopathologically unaffected normal thyroid tissue in the course of lobo- or thyroidetomies of thyroid lesions (4 goiters, 1 hyperthyroiditis, 1 PTC, and 1 FVPTC). Histopathological diagnosis and staging of thyroid lesions was performed by an experienced oncologic pathologist (JM) according to established criteria [19,20]. Five *BRAF*^wt^ PTCs were classified as micro PTCs (≤ 1 cm). This study was approved by the Research Ethics Committee of the King Abdulaziz University, Faculty of Medicine, #358-10, and the Institutional Review Board of the KFSH&RC, #IRB2010-07, and included written informed consent provided by the participants.

### DNA extraction and *BRAF* mutational screening

Genomic DNA from fresh-frozen samples was extracted using the QIAmp DNA tissue kit (Qiagen, Hilden, Germany). DNA concentration was measured with the Nanodrop ND-1000 spectrophotometer (Thermo Scientific, Wilmington, DE). Screening of the *BRAF* mutational hotspot region of exon 15 was performed as described earlier involving direct sequencing of PCR products spanning the region [5].

### RNA sample and array processing

Total RNA was extracted from freshly preserved thyroid tissue specimens using the Qiagen RNeasy Mini Kit (Qiagen, Hilden, Germany) including an on-column DNAse treatment according to manufacturer’s recommendations. Quality of the purified RNA was verified on an Agilent 2100 Bioanalyzer (Agilent Technologies, Palo Alto, CA). RNA integrity number for all evaluated samples was at least 5.0. RNA concentrations were determined using a NanoDrop ND-1000 spectrophotometer. Samples containing each 250 ng of RNA were processed using the Ambion WT Expression Kit (Life Technologies, Austin, TX) and the GeneChip WT Terminal Labeling and Controls Kit (Affymetrix, Santa Clara, CA) according to the manufacturers` recommendations. Fragmentation and endlabeling of samples were monitored by electrophoresis on 3 % agarose gels. Affymetrix GeneChip hybridization, wash and stain kits were utilized in subsequent processing steps. Hybridization mixtures containing each 5500 ng of cDNA were hybridized at 45°C for 17 hrs and 60 rpm to Affymetrix Human Gene 1.0 ST GeneChip arrays. Subsequent to wash and staining at the GeneChip Fluidics Station 450, the arrays were scanned with the GeneChip Scanner 3000 7G. Probe cell intensity data (CEL files) were generated using the GeneChip Command Console Software (AGCC). Human Gene 1.0 ST GeneChip arrays interrogate in total with a set of 764,885 probes 36.079 reference sequences (NCBI build 36).

### Gene Expression Analysis

CEL files were imported to Partek Genomics Suite version 6.6 (Partek Inc., MO) and a log-transformed data set of robust multi-array averaged (RMA), background-adjusted, and normalized values was generated. Non-annotated genes and multiple transcripts generated from the same gene were excluded from further analysis. Principal component analysis (PCA) was performed to assess quality as well as overall variance in gene expression between groups of samples. Analysis of Variance (ANOVA) was applied to generate a list of differentially expressed genes using a *p*-value with a false discovery rate (FDR) (Step up method) < 0.05 and a fold change > 2.0. Two dimensional average linkage hierarchical clustering was performed using Spearman’s correlation as a similarity matrix. The generated array data set complies with MIAME [21] and was submitted to NCBI’s Gene Expression Omnibus (GEO), accession number GSE54958.

### Functional network and pathway analysis

To define molecular networks and canonical pathways in differentially regulated gene sets, pathway analyses were performed by using Ingenuity Pathways Analysis (IPA) software (Ingenuity Systems, Redwood City, CA). For this purpose, statistically differentially expressed genes and their corresponding probe set ID, gene symbol as clone identifier, *p*-value and fold change were imported into IPA. The program identifies with its functional algorithms those biological functions, interacting drugs and/or diseases that are most significantly related to a differentially expressed gene set. The canonical pathway analysis identifies pathways that are most significantly related to the data set.

## Results

Demographic data of patients and histopathological criteria of *BRAF*^wt^ and *BRAF*^mut^ PTC are listed in Table 1. A gender shift towards females was observed in the *BRAF*^wt^ group and mean age was considerable lower in the *BRAF*^wt^ than in the *BRAF*^mut^ group (30.9 years *vs*. 45.9 years). Histopathological criteria including tumor size, LN involvement, tumor focality and tumor stage were comparably more unfavorable in *BRAF*^mut^ PTCs (Table 1).

**Table 1:** Demographic and clinicopathological features of *BRAF*^wt^ and *BRAF*^mut^ PTCs

Characteristics	PTCs	
Patient	*BRAF*^wt^ (N = 11)	*BRAF*^mut^ (N = 14)

age (year)		

Mean ± SD	30.9 ±12.2	45.9 ±16.4

Ë‚ 45	9	7

≥ 45	2	7

female	9	9

male	2	5

Tumor		

size ± SD (cm)	2.4±1.8	4.4±2.8

focal	6	3

multifocal	5	11

lymph node		

negative	5	3

positive	6	9

unknown	0	2

stage		

I	10	8

II	0	1

III	0	1

IV	1	3

unknown	0	1

### Expression *BRAF*^wt^*vs*. *BRAF*^mut^ PTCs

**Figure 1:**
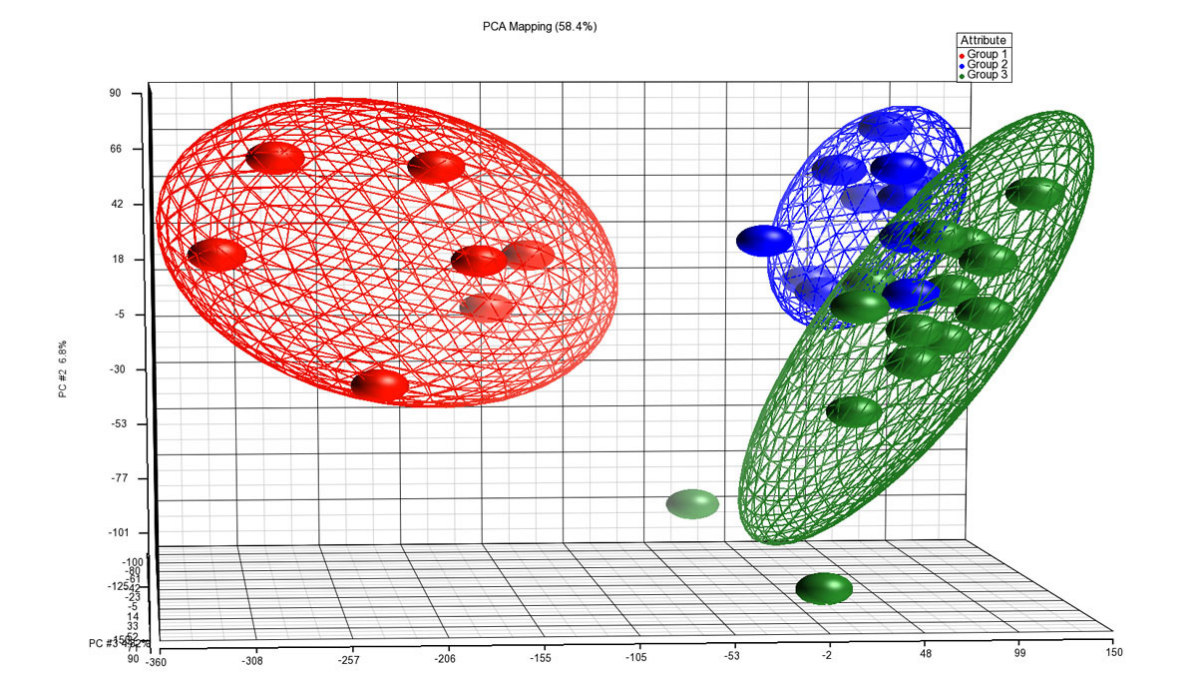
PCA scatter plot wherein each dot represents a sample with a group specific color. Distance between dots is a dimensional measure for the similarity of the respective expression profiles of the samples. Ellipsoids are a measure to visualize distance of relationships between samples of a group. Red, normal thyroid; blue, *BRAF*^wt^ PTCs, green, *BRAF*^mut^ PTCs.

**Figure 2:**
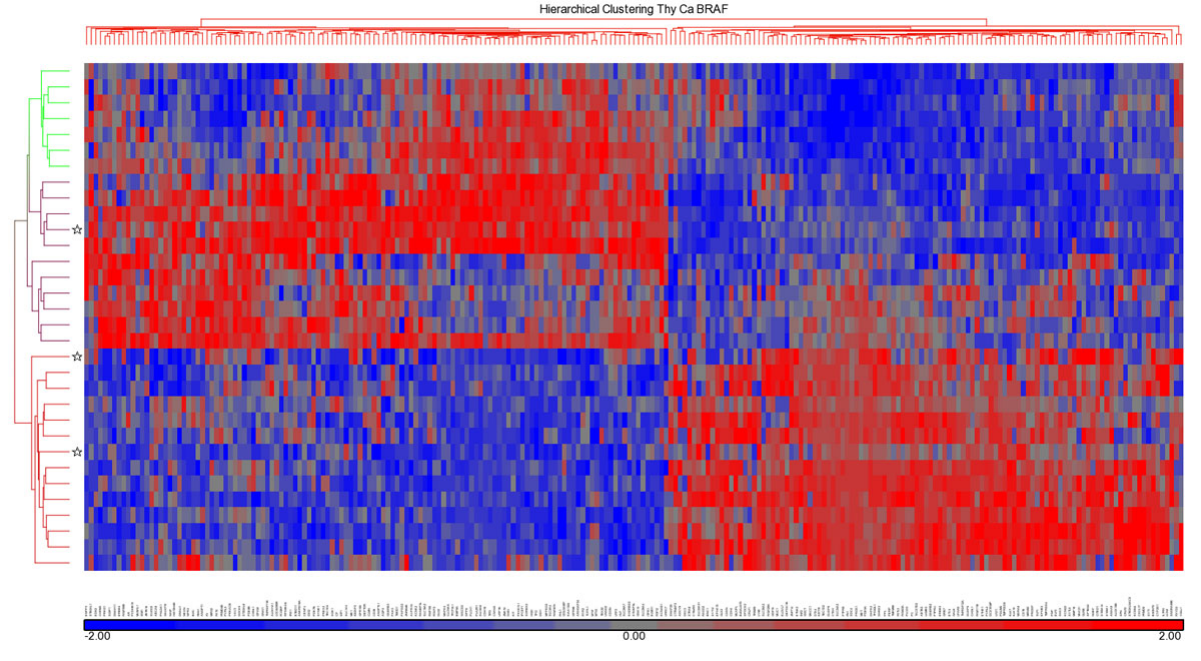
Hierarchial cluster analysis of 237 genes which were differentially expressed (FDR *p*-value ≤ 0.05 and fold change > 2.0) between *BRAF*^wt^ and *BRAF*^mut^ PTCs. Gene expression of TN samples is included in analysis. Color scheme for expression levels: red for comparably higher and blue for comparably lower expression. Color scheme for groups in the left sided branches: green for TN samples, purple for *BRAF*^wt^, and red for *BRAF*^mut^ PTCs. Stars mark samples from recurrence or local metastasis, and LN metastasis.

Employing whole-transcript microarrays (HuGene 1.0 ST) we compared expression profiles of 11 *BRAF*^wt^ PTCs with 14 *BRAF*^mut^ PTCs. Seven TN specimens were used as a reference for normal thyroid tissue expression which allowed us to identify differentially expressed genes between both PTC groups and TN samples. Three-D presentation of the PCA displays clustering of *BRAF*^wt^ PTCs, *BRAF*^mut^ PTCs and TN samples (Figure 1). We identified, after excluding non-annotated genes and multiple transcriptional isoforms, 237 annotated genes that were significantly differentially expressed (p-value with FDR <0.05 and a fold change >2) between *BRAF*^wt^ and *BRAF*^mut^ PTCs (Additional file 1). Of these genes, 127 were up-, and 110 were downregulated in *BRAF*^wt^ compared to *BRAF*^mut^ PTCs. The most significantly upregulated genes in *BRAF*^wt^ were *inositol 1*,*4*,*5-triphosphate receptor*, *type 1* (*ITPR1*), *hepatic leukemia factor* (*HLF*), *potassium voltage-gated channel*, *shaker-related subfamily*, *beta member 1* (*KCNAB1*), *engulfment and cell motility 1* (*ELMO1*), *Rho GTPase activating protein 24* (*ARHGAP24*), *thyroid peroxidase* (*TPO*) and *solute carrier family 4*, *sodium bicarbonate cotransporter*, *member* 4 (*SLC4A4*). The most significantly downregulated genes in *BRAF*^wt^ PTCs were *dendrocyte expressed seven transmembrane protein* (*DCSTAMP*), *ladinin 1* (*LAD1*), *keratin 19* (*KRT19*), *chromosome 19 open reading frame 33* (*C19orf33*), *poliovirus receptor-related 4* (*PVRL4*), *EPH receptor A10* (*EPHA10*), and *TBC1 domain family*, *member 2* (*TBC1D2*). The list of 237 genes contained one microRNA (*MIR492*) that was downregulated and one microRNA (*MIR32*) that was upregulated in *BRAF*^wt^*vs*. *BRAF*^mut^ PTCs. Additional file 1 contains also the cellular/extracellular location, function of gene products as well as a selection of drugs known to interact with a gene product. Hierarchical cluster analysis of the 237 genes separates both PTC groups according to their differential gene expression (Figure 2). TN samples were included in this data set to provide normal expression values.

### Expression PTCs *vs*. TN samples

Comparison of genes differentially expressed between *BRAF*^wt^ and TN samples revealed a set of 8249 genes and between *BRAF*^mut^ and TN samples a set of 8836 genes. To identify genes of interests, e.g. conceivable immunohistochemistry markers, we selected those genes which are differentially expressed between all three groups (Table 2). In sum, 32 genes met the statistical criteria and the vast majority revealed the comparatively highest expression in *BRAF*^mut^ PTCs.

**Table 2:** Differentially expressed genes between *BRAF*^wt^ and *BRAF*^mut^ PTCs and TN samples

Gene name	Gene symbol	*BRAF*^wt^ PTC *vs. BRAF*^mut^ PTC		*BRAF*^wt^ PTC *vs*. TN		*BRAF*^mut^ PTC *vs*. TN	
		
		FC^1^	*P*	FC^1^	*P*	FC^1^	*P* ^2^
keratin 19	KRT19	-4.39	1.3E-09	2.37	2.0E-04	10.43	8.5E-13

potassium voltage-gated channel, shaker-related subfamily, beta member 1	KCNAB1	7.66	6.4E-09	3.23	5.5E-04	-2.37	5.7E-03

solute carrier family 26, member 4	SLC26A4	7.59	4.3E-08	2.14	2.9E-02	-3.55	4.0E-04

TBC1 domain family, member 2	TBC1D2	-2.39	4.4E-08	2.71	1.0E-07	6.48	3.3E-14

tumor protein D52-like 1	TPD52L1	-2.46	3.2E-07	2.22	3.6E-05	5.46	1.1E-11

TP53 apoptosis effector	PERP	-2.09	3.6E-07	2.97	6.9E-09	6.22	1.6E-14

Periplakin	PPL	-2.53	1.0E-06	2.92	1.9E-06	7.41	2.3E-12

growth differentiation factor 15	GDF15	-5.08	1.5E-06	2.41	1.1E-02	12.23	6.8E-09

deltex homolog 4 (Drosophila)	DTX4	-3.31	3.5E-06	3.28	5.6E-05	10.86	8.4E-11

death-associated protein kinase 2	DAPK2	-2.45	1.0E-05	2.78	2.1E-05	6.83	7.6E-11

ethanolamine kinase 2	ETNK2	-2.51	1.9E-05	2.02	3.0E-03	5.07	1.3E-08

solute carrier family 34 (sodium phosphate), member 2	SLC34A2	-4.75	2.2E-05	8.86	2.2E-06	42.09	1.9E-11

transmembrane protein 98	TMEM98	-2.09	2.8E-05	2.24	9.8E-05	4.69	6.2E-10

gamma-glutamylcyclotransferase	GGCT	-2.52	3.1E-05	2.48	3.7E-04	6.26	2.3E-09

purine nucleoside phosphorylase	PNP	-3.55	3.4E-05	2.48	6.6E-03	8.82	4.7E-08

protein S (alpha)	PROS1	-2.80	3.7E-05	5.78	1.4E-07	16.18	2.8E-12

FERM domain containing 4B	FRMD4B	2.50	3.7E-05	5.04	7.3E-08	2.01	3.1E-03

mucin 1, cell surface associated	MUC1	-2.29	5.6E-05	2.23	6.8E-04	5.12	6.6E-09

protein tyrosine phosphatase, receptor type, E	PTPRE	-2.22	6.0E-05	2.45	1.3E-04	5.43	1.5E-09

gamma-aminobutyric acid (GABA) A receptor, beta 2	GABRB2	-4.73	8.6E-05	2.82	1.7E-02	13.32	3.0E-07

metastasis associated in colon cancer 1	MACC1	-2.34	1.2E-04	2.15	2.3E-03	5.02	4.0E-08

Cbp/p300-interacting transactivator, with Glu/Asp-rich carboxy-terminal domain, 1	CITED1	-3.58	3.4E-04	2.56	1.8E-02	9.17	1.1E-06

laminin, beta 3	LAMB3	-3.13	3.4E-04	2.46	1.2E-02	7.70	6.7E-07

syndecan 4	SDC4	-2.22	4.3E-04	4.25	1.6E-06	9.45	1.2E-10

family with sequence similarity 129, member A	FAM129A	-2.21	4.3E-04	2.27	1.9E-03	5.03	9.8E-08

fibronectin 1	FN1	-2.60	4.9E-04	10.31	8.2E-09	26.78	1.5E-12

phosphodiesterase 5A, cGMP-specific	PDE5A	-2.82	7.1E-04	6.06	6.7E-06	17.11	6.5E-10

dual specificity phosphatase 5	DUSP5	-2.11	7.9E-04	2.16	3.1E-03	4.57	2.8E-07

chitinase 3-like 1 (cartilage glycoprotein-39)	CHI3L1	-4.45	8.5E-04	3.50	1.4E-02	15.59	1.8E-06

cathepsin H	CTSH	-2.63	9.2E-04	5.31	1.1E-05	13.97	1.2E-09

trefoil factor 3 (intestinal)	TFF3	3.81	1.5E-05	-2.86	2.6E-02	-10.92	5.4E-06

met proto-oncogene (hepatocyte growth factor receptor)	MET	-2.27	2.7E-11	8.93	1.3E-08	20.25	4.6E-12

^1^FC, fold change; -, downregulated in *BRAF*^wt^ PTCs vs. *BRAF*^mut^ PTCs, downregulated in *BRAF*^wt^ PTCs vs. TN, and downregulated in *BRAF*^mut^ PTCs vs. TN; ^2^p-value with FDR < 0.05 and fold change cutoff > 2

### Gene networks and canonical pathways

**Figure 3:**
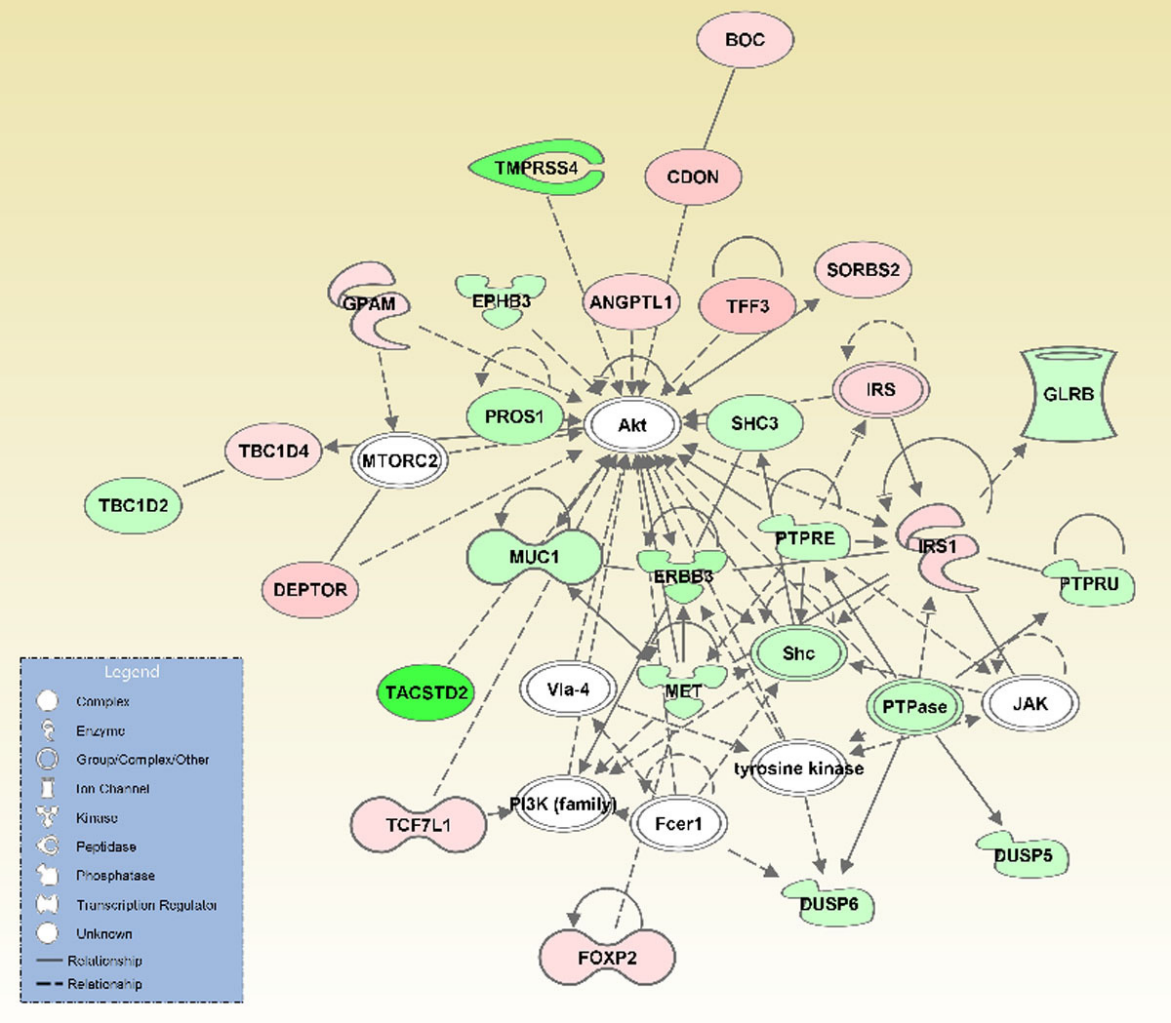
A molecular network including 12 overexpressed (red) and 16 underexpressed (green) molecules in *BRAF*^wt^ compared to *BRAF*^mut^ PTCs. The relationships between molecules were conveyed by IPA functions on basis of the set 237 differentially expressed genes in *BRAF*^wt^*vs*. *BRAF*^mut^ PTCs. Gene names in Additional file 1.

**Figure 4:**
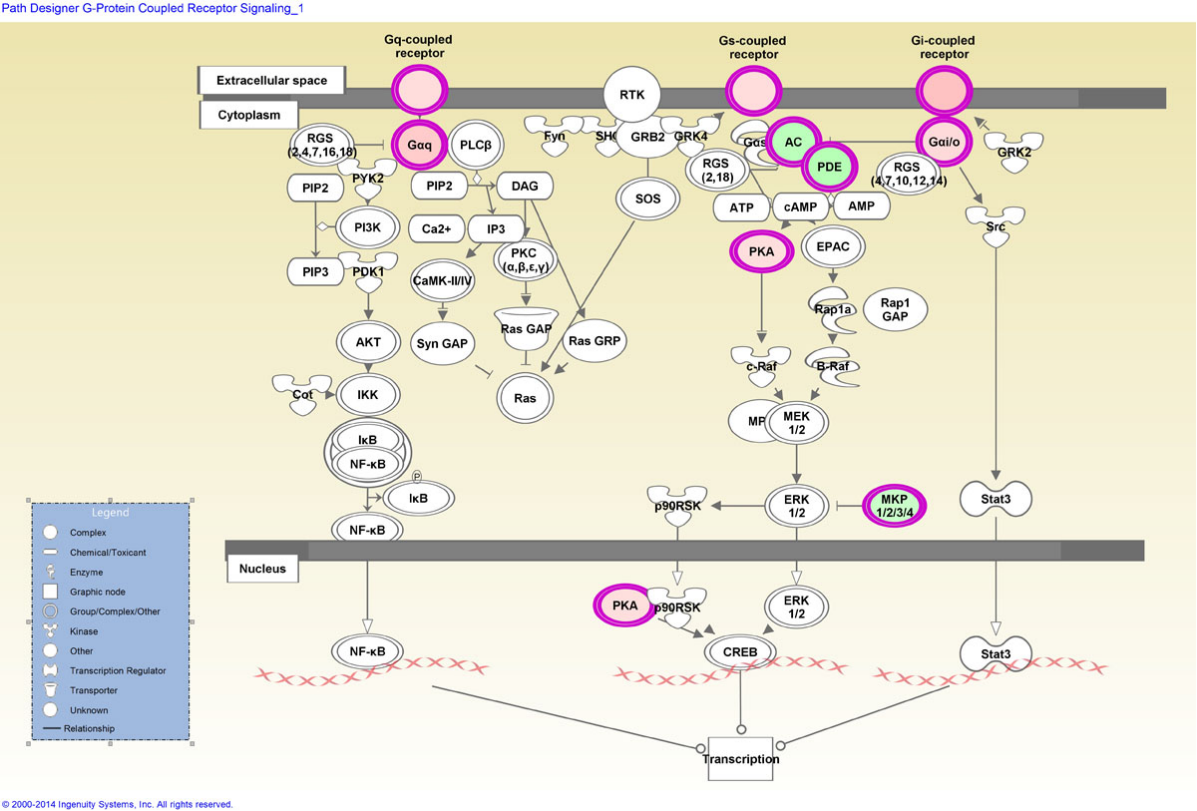
The G protein-coupled receptor pathway is involved in transmembrane signaling and diverse physiological functions including hormone signaling, regulation of cell contraction, cell migration, growth and differentiation. Members of the G protein-coupled receptors, G protein alpha q, G protein alpha i/o, and PKA are upregulated (red) whereas members of MAPK phosphatases (MKP), phosphodiesterases (PDE) and adenylate cyclases (AC) are downregulated (green) in *BRAF*^wt^ compared to *BRAF*^mut^ PTCs.

Most significant network functions identified by IPA algorithms and associated with the set of 237 differentially expressed genes were involved in cell signaling, cancer, and cellular development (Figure 3). This comprehensive network includes 12 molecules which are overexpressed and 16 molecules which are underexpressed in *BRAF*^wt^ compared to *BRAF*^mut^ PTCs. The most significantly associated canonical pathway related to the differentially expressed set of 237 genes is the proteinkinase A (PKA) signaling cascade (Additional file 2). The PKA pathway is involved in second messenger signaling and it is stimulated by upstream cascades including the G protein-coupled receptor pathway (Figure 4). The most significant pathways with thyroid specific functions were the thyroid hormone metabolic and thyronamine/iodothyronamine metabolic pathways.

## Discussion

We performed one of the first studies using whole transcript microarrays to compare expression profiles solely in conventional *BRAF*^wt^ and *BRAF*^mut^ PTCs. Comparison to studies including different histological subtypes of PTCs may result in detecting a lower number of common genes. An expression array study including histological variants of PTC identified, on basis of enhanced stringent threshold criteria, over 80 up- and downregulated genes in the *BRAF*^mut^ group in comparison to PTCs with either a RAS mutation or a RET/PTC rearrangement [15] and the 40 most up- and downregulated genes have an overlap of ~40 % to our list of 237 differentially expressed genes.

### Selected differentially expressed molecules

*DCSTAMP*, also known as TM7SF4, has been previously identified as one of the most overexpressed genes in *BRAF*^mut^ PTC compared to *BRAF*^wt^ PTC as well in PTC with undetermined mutational status compared to normal thyroid tissue [15,22,23]. It has been supposed that *DCSTAMP* expression is an immune response related to *BRAF*^mut^ tumors [15]. DCSTAMP contains signature motifs owned by the family of transmembrane serine proteases and it exhibits degradation activity against extracellular matrix proteins. One of the function of the hepatic leukemia factor (HLF) as a transcription factor is to mediate thyroid hormone activation from the thyroid hormone receptor/retinoid X receptor heterodimer to the *hypoxia-inducible factor* (*HIF-1alpha*) [24]. The function of *C19orf33*, also known as H2RSP (Hepatocyte growth factor activator inhibitor type 2-related small protein) is virtually unknown. An enhanced expression with higher levels in LN positive tumors has been observed for C19orf33 in colorectal adenocarcinoma cells at the invasive front [25]. The voltage gated channel molecule KCNAB1 exhibits diverse functions including electrolyte transport, and insulin secretion. Downregulation of *KCNAB1* expression has been identified in follicular thyroid carcinomas compared to benign follicular adenomas [26]. ELMO1 functionally interacts with dedicator of cytokinesis 1 (DOCK1) that promotes Rac guanine exchange factor (GEF) activity for Rac proteins of the Rho GTPases family. GTP-loaded Rac proteins initiate downstream pathways that promote cell elongation, migration, and cytoskeleton remodeling [27,28]. The active ELMO1/DOCK1 complex is anchored via phosphoinositides to the membrane. In our study, the *DOCK1* related *DOCK5* gene was identified as a 2-fold upregulated molecule in *BRAF*^wt^ compared to *BRAF*^mut^ PTCs. The *leucine-rich repeat and immunoglobulin-like domain-containing nogo receptor-interacting protein 2* (*LINGO2*) encodes a single-pass type I membrane protein that is primarily expressed during development in cells adjunct to the epithelial lining of the olfactory pit and in adult brain [29]. In our study, *LINGO2* was downregulated in *BRAF*^wt^ PTCs and even more in *BRAF*^mut^ PTCs compared to TN samples which may imply a tumor suppressor function for this molecule. Higher expression levels of the ERK1/2-specific cytoplasmic dual specificity phosphatase 6 (DUSP6) in comparison to benign and normal thyroid cells has been previously associated with PTC, especially with advanced thyroid carcinomas [30,31]. In our study, *DUSP6* was 2.1 times higher expressed in *BRAF*^mut^ than in *BRAF*^wt^ PTCs assuming that the ERK1/2 related pathway is frequently more utilized in the *BRAF*^mut^ group. *MET* overexpression in thyroid cancer has been identified in a number of studies and this molecule was 2.3-fold higher expressed in our study in *BRAF*^mut^ compared to *BRAF*^wt^ PTCs which is in accordance with another survey [15] (Table 2).

### Second messenger molecules

ITPR1 is an intracellular receptor for inositol 1,4,5-trisphosphate (IP3) and implicated in the thyroid hormone synthesis pathway. The receptor mediates calcium release from the endoplasmic reticulum upon stimulation by IP3. Downregulation of *ITPR1* has been demonstrated in thyroid cancer in comparison to non-malignant thyroid tissue in a number of studies [32-35]. Among the members of the phospholipase C (PLC) family, *PLCH1* was upregulated in *BRAF*^wt^ PTCs whereas *PLCD3* was downregulated. PLC molecules hydrolyze phosphatidylinositol 4,5-bisphosphate to generate the second messenger diacylglycerol and IP3.

### Thyroid hormone pathway molecules

*LAD1* has been identified as an overexpressed gene in *BRAF*^mut^ thyroid carcinomas compared to those with a RET/PTC rearrangement [18]. *Lad1* expression is regulated by the glucocorticoid receptor (GR) and requires for induction the GR coactivators thyroid hormone receptor associated protein 220 (MED1) and the thyroid hormone receptor associated protein 170 (MED14) [36]. Downregulation of *TPO* has been considered in a number of studies comparing thyroid carcinoma with thyroid carcinomas with benign thyroid tumors or normal thyroid samples [32,33,37,38]. However, downregulation of *TPO* and the sodium iodine symporter genes as been previously associated with *BRAF*^mut^ PTCs in comparison to PTCs with a RET/PTC rearrangement [39]. This is in line with observations that *BRAF*^mut^ tumors are refractory for radioactive iodine ablation due to downregulation of thyroid hormone biosynthesis pathways [40]. *TPO* was in our study 13-fold downregulated in *BRAF*^mut^ compared to *BRAF*^wt^ PTCs. In addition, other genes involved in thyroid hormone biosynthesis pathway including the *solute carrier family-5 member-8* (*SLC5A8*), *solute carrier family 26* (*anion exchanger*), *member 4* (*SLC26A4*), *deiodinase*, *iodothyronine*, *type I* (*DIO1*) and *deiodinase*, *iodothyronine*, *type II* (*DIO2*) were downregulated in our set of *BRAF*^mut^ PTCs. Other members of the SLC family that were downregulated in *BRAF*^mut^ PTCs were *SLC1A3*, *SLC4A4*, *SLC16A2*, and *SLC26A7* whereas *SLC22A31*, *SLC30A2*, and *SLC34A2* were downregulated in *BRAF*^wt^ PTCs.

### *ERBB3* and *ERRB4* genes

Two of the four structurally related receptor tyrosine kinases ErbB, namely *ERBB3* and *ERBB4*, were differentially expressed in the two PTC groups, i.e. *ERBB3* was downregulated and *ERBB4* upregulated in *BRAF*^wt^ PTCs (Additional file 1). Of notice, *ERBB4* was also lower expressed in BRAF^mut^ PTCs compared to TN samples (p = 0.0035) (data not shown). Oncogenic functions of ERBB3 and ERBB4, which can form heterodimers and signal through the PI3K/AKT signaling pathway have not been elucidated yet in detail in relation to the *BRAF* mutational status in thyroid cancer [41-43]; however, decreased expression of *ERBB4* in PTC *vs*. normal thyroid tissue has been demonstrated in a RT-PCR study whereas *ERBB2* and *ERBB3* expression was shown to be increased [44]. On the protein level, a tissue microarray study in proliferative thyroid lesions found a correlation of ERBB3 expression with LN metastasis whereas ERBB4 expression correlated with lower tumor stage [45]. A possible link of *ERBB3* to apoptosis can be deduced from a functional *in vivo* study wherein deletion of *ERBB3* in mouse intestinal epithelium induced tumor-specific cell death [46].

### MicroRNAs

The most significantly downregulated microRNAs in *BRAF*^wt^ compared to *BRAF*^mut^ PTCs was *mir492* and the most upregulated one was *mir32*. Overexpression of *mir492* has been previously linked to progressive hepatoblastoma and tumorigenesis of retinoblastoma [47,48]. Of notice, *mir492* is processed from the *KRT19* gene [47] and both are higher expressed. A possible target for miR492 is the 3`UTR of KRT19 [49] which is 4.4-fold higher expressed in *BRAF*^mut^ PTCs compared to *BRAF*^wt^ PTCs suggesting an accumulation of the KRT19 mRNA. Another possible target of miR492 is the thyroid hormone receptor-associated protein 3 (THRAP3) mRNA that harbors two predicted target sites for miR492 [49] and that is significantly downregulated in our case series by approximately 3-fold in both, *BRAF*^wt^ and *BRAF*^mut^ PTCs compared to TN samples. Upregulation of *mir32* in thyroid cancer *vs*. benign thyroid tumors has been detected in a microarray study; however functional implication of this microRNA in PTC is not known yet [50].

In summary, our microarray expression study provides a detailed overview of differentially expressed genes, networks, and pathways between *BRAF*^wt^ and *BRAF*^mut^ PTCs that gain interest for basic molecular genetics and translational studies in PTCs.

## List of abbreviations

*BRAF*^wt^: *BRAF* wild type; *BRAF*^mut^: *BRAF* mutant; FVPTC: follicular variant of PTC; LN: lymph node; PTC: papillary thyroid carcinoma; TN: normal thyroid.

## Competing interests

The authors declare that they have no competing interests.

## Authors’ contributions

JM, EH, MG, and MHQ made substantial contributions to conception and design of the study. RA, AA, and MA processed expression arrays, performed *BRAF* mutational analysis, and were involved in data interpretation. KG, FM, and OAH were responsible for surgeries, oversight of clinical databases and contributed to the conception and design of the study. JM performed histological examinations. SK and HJS performed data analysis. HJS had general oversight of the study. HJS, JM, and MHQ interpreted data and drafted the manuscript. All authors read and approved the final manuscript.

## Supplementary Material

Additional file 1 The 237 most differentially expressed genes in *BRAF*^wt^ vs. *BRAF*^mut^ papillary thyroid carcinomas. Click here for file

Additional file 2 The canonical PKA pathway is a second messenger cascade and involved in diverse functions as growth, development, metabolism, DNA replication/recombination, DNA repair and cellular organization. A number of molecules including members of PKA, ryanodine receptors (RYR), inositol trisphosphate receptors (IP3R), and lymphoid enhancing factors/T-cell factors (TCF/LEF) are upregulated (red) and number of molecules including members of phospholipases C (PLC), 14-3-3 proteins, and protein tyrosine phosphatases (PTP) are downregulated (green) in *BRAF*^wt^ compared to *BRAF*^mut^ PTCs. Click here for file

## Declarations

Publication charges for this article have been funded by the Center of Excellence in Genomic Medicine Research (CEGMR), King Abdulaziz University, Jeddah, Kingdom of Saudi Arabia.

This article has been published as part of *BMC Genomics* Volume 16 Supplement 1, 2015: Selected articles from the 2nd International Genomic Medical Conference (IGMC 2013): Genomics. The full contents of the supplement are available online at http://www.biomedcentral.com/bmcgenomics/supplements/16/S1
