# Supplementary material for: Effect of BRAF mutational status on expression profiles in conventional papillary thyroid carcinomas
Source: BMC Genomics. 2015 Jan 15;16(Suppl 1):S6. doi: 10.1186/1471-2164-16-S1-S6 (PMC4315163; doi:10.1186/1471-2164-16-S1-S6)
Supplement: Additional file 1 — The 237 most differentially expressed genes in BRAFwt vs. BRAFmut papillary thyroid carcinomas. [file 1471-2164-16-S1-S6-S1.docx]

| **Additional file 1: The 237 most differentially expressed genes in *BRAF*^wt^ vs. *BRAF*^mut^ papillary thyroid carcinomas** | | | | | | |
| --- | --- | --- | --- | --- | --- | --- |
| ***P*** | **Fold^1^ change** | **Gene symbol** | **Entrez gene name** | **Location^2^** | **Function^2^** | **Interacting drugs** |
| 4.05E-15 | -52.685 | DCSTAMP | dendrocyte expressed seven transmembrane protein | plasma membrane | ns |  |
| 3.77E-11 | 4.932 | ITPR1 | inositol 1,4,5-trisphosphate receptor, type 1 | cytoplasm | ion channel |  |
| 7.06E-10 | -2.808 | LAD1 | ladinin 1 | extracellular space | ns |  |
| 1.33E-09 | -4.390 | KRT19 | keratin 19 | cytoplasm | ns |  |
| 3.07E-09 | 3.925 | HLF | hepatic leukemia factor | nucleus | transcription regulator |  |
| 4.54E-09 | -2.310 | C19orf33 | chromosome 19 open reading frame 33 | nucleus | ns |  |
| 6.41E-09 | 7.664 | KCNAB1 | potassium voltage-gated channel, shaker-related subfamily, beta member 1 | plasma membrane | ion channel |  |
| 6.83E-09 | 3.788 | ELMO1 | engulfment and cell motility 1 | cytoplasm | ns |  |
| 1.01E-08 | -2.772 | PVRL4 | poliovirus receptor-related 4 | plasma membrane | ns |  |
| 1.21E-08 | -2.249 | EPHA10 | EPH receptor A10 | plasma membrane | transmembrane receptor |  |
| 1.90E-08 | 2.530 | ARHGAP24 | Rho GTPase activating protein 24 | cytoplasm | ns |  |
| 2.85E-08 | 13.346 | TPO | thyroid peroxidase | plasma membrane | enzyme | propylthiouracil, carbimazole, methimazole |
| 3.37E-08 | 6.115 | SLC4A4 | solute carrier family 4 (sodium bicarbonate cotransporter), member 4 | plasma membrane | transporter |  |
| 4.30E-08 | 7.590 | SLC26A4 | solute carrier family 26 (anion exchanger), member 4 | plasma membrane | transporter |  |
| 4.40E-08 | -2.388 | TBC1D2 | TBC1 domain family, member 2 | cytoplasm | ns |  |
| 4.41E-08 | 2.312 | GALNT10 | UDP-N-acetyl-alpha-D-galactosamine:polypeptide N-acetylgalactosaminyltransferase 10 (GalNAc-T10) | cytoplasm | enzyme |  |
| 4.49E-08 | -8.836 | KLK7 | kallikrein-related peptidase 7 | extracellular space | peptidase |  |
| 4.96E-08 | 2.919 | LINGO2 | leucine rich repeat and Ig domain containing 2 | extracellular space | ns |  |
| 6.56E-08 | -4.315 | B3GNT3 | UDP-GlcNAc:betaGal beta-1,3-N-acetylglucosaminyltransferase 3 | cytoplasm | enzyme |  |
| 6.63E-08 | -2.099 | SNAP25 | synaptosomal-associated protein, 25kDa | plasma membrane | transporter | botulinum toxin type A |
| 6.90E-08 | 6.319 | HGD | homogentisate 1,2-dioxygenase | cytoplasm | enzyme |  |
| 7.18E-08 | -3.108 | PLCD3 | phospholipase C, delta 3 | cytoplasm | enzyme |  |
| 7.64E-08 | -3.366 | TMPRSS6 | transmembrane protease, serine 6 | plasma membrane | peptidase |  |
| 9.45E-08 | 22.756 | DIO1 | deiodinase, iodothyronine, type I | cytoplasm | enzyme | propylthiouracil |
| 1.00E-07 | -2.221 | SPTBN2 | spectrin, beta, non-erythrocytic 2 | cytoplasm | ns |  |
| 1.04E-07 | 3.467 | PRKG1 | protein kinase, cGMP-dependent, type I | cytoplasm | kinase |  |
| 1.08E-07 | -2.171 | GLS2 | glutaminase 2 (liver, mitochondrial) | cytoplasm | enzyme |  |
| 1.31E-07 | 2.576 | ST3GAL6 | ST3 beta-galactoside alpha-2,3-sialyltransferase 6 | cytoplasm | enzyme |  |
| 1.47E-07 | -2.620 | CYP2S1 | cytochrome P450, family 2, subfamily S, polypeptide 1 | cytoplasm | enzyme |  |
| 1.52E-07 | 4.608 | FHL1 | four and a half LIM domains 1 | cytoplasm | ns |  |
| 1.56E-07 | 3.101 | SGIP1 | SH3-domain GRB2-like (endophilin) interacting protein 1 | cytoplasm | ns |  |
| 2.26E-07 | 5.181 | LRP2 | low density lipoprotein receptor-related protein 2 | plasma membrane | transporter |  |
| 2.94E-07 | -2.245 | PC | pyruvate carboxylase | cytoplasm | enzyme |  |
| 3.00E-07 | -3.011 | ERBB3 | v-erb-b2 avian erythroblastic leukemia viral oncogene homolog 3 | plasma membrane | kinase | sapitinib |
| 3.24E-07 | -2.458 | TPD52L1 | tumor protein D52-like 1 | cytoplasm | ns |  |
| 3.40E-07 | 2.491 | GPR98 | G protein-coupled receptor 98 | plasma membrane | G-protein coupled receptor |  |
| 3.56E-07 | -2.092 | PERP | PERP, TP53 apoptosis effector | plasma membrane | ns |  |
| 3.88E-07 | -7.429 | TACSTD2 | tumor-associated calcium signal transducer 2 | plasma membrane | ns |  |
| 4.19E-07 | -2.343 | MLXIPL | MLX interacting protein-like | nucleus | transcription regulator |  |
| 6.38E-07 | 2.066 | DOCK5 | dedicator of cytokinesis 5 | cytoplasm | ns |  |
| 7.02E-07 | -2.294 | MIR492 | micro RNA 492 | cytoplasm | micro RNA |  |
| 7.14E-07 | 2.697 | PREX2 | phosphatidylinositol-3,4,5-trisphosphate-dependent Rac exchange factor 2 | cytoplasm | ns |  |
| 7.82E-07 | 3.425 | CDON | cell adhesion associated, oncogene regulated | plasma membrane | ns |  |
| 8.33E-07 | 5.884 | MPPED2 | metallophosphoesterase domain containing 2 | ns | ns |  |
| 8.65E-07 | 2.421 | IRS1 | insulin receptor substrate 1 | cytoplasm | enzyme |  |
| 9.26E-07 | -3.410 | PDLIM4 | PDZ and LIM domain 4 | plasma membrane | ns |  |
| 1.02E-06 | -2.533 | PPL | periplakin | cytoplasm | ns |  |
| 1.07E-06 | 2.866 | RYR2 | ryanodine receptor 2 (cardiac) | plasma membrane | ion channel |  |
| 1.09E-06 | -2.227 | PTPRU | protein tyrosine phosphatase, receptor type, U | plasma membrane | phosphatase |  |
| 1.55E-06 | -5.077 | GDF15 | growth differentiation factor 15 | extracellular Space | growth factor |  |
| 1.59E-06 | 6.776 | SLC5A8 | solute carrier family 5 (sodium/monocarboxylate cotransporter), member 8 | plasma membrane | transporter |  |
| 1.64E-06 | 5.055 | STXBP5L | syntaxin binding protein 5-like | cytoplasm | ns |  |
| 1.67E-06 | -2.535 | FAM43A | family with sequence similarity 43, member A | ns | ns |  |
| 1.97E-06 | 2.066 | GPAM | glycerol-3-phosphate acyltransferase, mitochondrial | cytoplasm | enzyme |  |
| 2.11E-06 | -3.308 | CDSN | corneodesmosin | plasma membrane | ns |  |
| 2.12E-06 | 2.131 | ME1 | malic enzyme 1, NADP(+)-dependent, cytosolic | cytoplasm | enzyme |  |
| 2.14E-06 | 5.184 | AQP4 | aquaporin 4 | plasma membrane | transporter |  |
| 2.17E-06 | 2.748 | SLC1A3 | solute carrier family 1 (glial high affinity glutamate transporter), member 3 | plasma membrane | transporter | riluzole |
| 2.20E-06 | -2.501 | ABTB2 | ankyrin repeat and BTB (POZ) domain containing 2 | ns | ns |  |
| 2.38E-06 | -2.312 | GRB7 | growth factor receptor-bound protein 7 | plasma membrane | ns |  |
| 2.43E-06 | 2.035 | GOLGA8IP | golgin A8 family, member I | ns | ns |  |
| 2.47E-06 | -2.066 | INF2 | inverted formin, FH2 and WH2 domain containing | cytoplasm | ns |  |
| 2.51E-06 | 3.338 | GNA14 | guanine nucleotide binding protein (G protein), alpha 14 | plasma membrane | enzyme |  |
| 2.63E-06 | 6.872 | KIAA1324 | KIAA1324 | plasma membrane | ns |  |
| 2.85E-06 | 2.467 | HIST2H4B | histone cluster 1, H4a | nucleus | ns |  |
| 3.32E-06 | -2.877 | STRA6 | stimulated by retinoic acid 6 | plasma membrane | ns |  |
| 3.54E-06 | -3.313 | DTX4 | deltex homolog 4 (Drosophila) | cytoplasm | ns |  |
| 3.94E-06 | 2.861 | RANBP3L | RAN binding protein 3-like | ns | ns |  |
| 4.14E-06 | -2.249 | FSTL3 | follistatin-like 3 (secreted glycoprotein) | extracellular space | ns |  |
| 4.29E-06 | 4.138 | DPP6 | dipeptidyl-peptidase 6 | plasma membrane | peptidase |  |
| 4.40E-06 | 2.921 | CGNL1 | cingulin-like 1 | plasma membrane | ns |  |
| 4.54E-06 | 2.866 | VAV3 | vav 3 guanine nucleotide exchange factor | extracellular space | cytokine |  |
| 5.09E-06 | -2.245 | EPHB3 | EPH receptor B3 | plasma membrane | kinase |  |
| 5.11E-06 | -2.471 | ELF3 | E74-like factor 3 (ets domain transcription factor, epithelial-specific ) | nucleus | transcription regulator |  |
| 5.28E-06 | 3.121 | DNAH11 | dynein, axonemal, heavy chain 11 | cytoplasm | enzyme |  |
| 5.46E-06 | 2.445 | SYNE1 | spectrin repeat containing, nuclear envelope 1 | nucleus | ns |  |
| 5.67E-06 | 2.855 | ERBB4 | v-erb-b2 avian erythroblastic leukemia viral oncogene homolog 4 | plasma membrane | kinase | BMS-599626, afatinib |
| 5.90E-06 | 5.456 | SLC26A7 | solute carrier family 26 (anion exchanger), member 7 | plasma membrane | transporter |  |
| 6.08E-06 | 2.960 | PLCH1 | phospholipase C, eta 1 | cytoplasm | enzyme |  |
| 6.11E-06 | 2.028 | RNF150 | ring finger protein 150 | ns | peptidase |  |
| 6.41E-06 | 4.948 | BMP8A | bone morphogenetic protein 8a | ns | ns |  |
| 6.57E-06 | -5.136 | LCN2 | lipocalin 2 | extracellular space | transporter |  |
| 6.63E-06 | -2.473 | NGEF | neuronal guanine nucleotide exchange factor | cytoplasm | ns |  |
| 6.80E-06 | -2.265 | BNC1 | basonuclin 1 | nucleus | transcription regulator |  |
| 6.98E-06 | 3.219 | DEPTOR | DEP domain containing MTOR-interacting protein | ns | ns |  |
| 8.62E-06 | 2.737 | WSCD2 | WSC domain containing 2 | ns | ns |  |
| 9.20E-06 | 2.152 | CLMN | calmin (calponin-like, transmembrane) | cytoplasm | ns |  |
| 9.24E-06 | -2.009 | SYT12 | synaptotagmin XII | plasma membrane | transporter |  |
| 9.70E-06 | -3.451 | DEPDC1B | DEP domain containing 1B | cytoplasm | ns |  |
| 9.97E-06 | 2.370 | FGF12 | fibroblast growth factor 12 | extracellular space | ns |  |
| 9.99E-06 | -2.454 | DAPK2 | death-associated protein kinase 2 | cytoplasm | kinase |  |
| 1.01E-05 | 2.517 | SORBS2 | sorbin and SH3 domain containing 2 | plasma membrane | ns |  |
| 1.13E-05 | -5.823 | TMPRSS4 | transmembrane protease, serine 4 | plasma membrane | peptidase |  |
| 1.22E-05 | 2.095 | AR | androgen receptor | nucleus | ligand-dependent nuclear receptor | estradiol valerate/ testosterone enanthate, estradiol and nss |
| 1.26E-05 | -3.492 | ST6GALNAC5 | ST6 (alpha-N-acetyl-neuraminyl-2,3-beta-galactosyl-1,3)-N-acetylgalactosaminide alpha-2,6-sialyltransferase 5 | cytoplasm | enzyme |  |
| 1.45E-05 | -3.753 | CXCL17 | chemokine (C-X-C motif) ligand 17 | ns | ns |  |
| 1.48E-05 | 2.061 | FAT4 | FAT atypical cadherin 4 | ns | ns |  |
| 1.50E-05 | -2.322 | MXRA8 | matrix-remodelling associated 8 | ns | ns |  |
| 1.60E-05 | -2.019 | ETHE1 | ethylmalonic encephalopathy 1 | cytoplasm | enzyme |  |
| 1.93E-05 | -2.512 | ETNK2 | ethanolamine kinase 2 | cytoplasm | kinase |  |
| 2.20E-05 | -4.753 | SLC34A2 | solute carrier family 34 (type II sodium/phosphate contransporter), member 2 | plasma membrane | transporter |  |
| 2.54E-05 | 3.624 | FAM167A | family with sequence similarity 167, member A | ns | ns |  |
| 2.62E-05 | 2.475 | AVPR1A | arginine vasopressin receptor 1A | plasma membrane | G-protein coupled receptor | conivaptan, AVP, lypressin |
| 2.67E-05 | 2.148 | PRKACB | protein kinase, cAMP-dependent, catalytic, beta | cytoplasm | kinase |  |
| 2.77E-05 | -2.095 | TMEM98 | transmembrane protein 98 | cytoplasm | ns |  |
| 2.81E-05 | 4.082 | IGF1 | insulin-like growth factor 1 (somatomedin C) | extracellular space | growth factor |  |
| 2.98E-05 | 2.180 | CHRM3 | cholinergic receptor, muscarinic 3 | plasma membrane | G-protein coupled receptor | fesoterodine, ABT-089, aclidinium, atropine/ edrophonium, and nss |
| 3.09E-05 | 2.340 | SGK1 | serum/glucocorticoid regulated kinase 1 | cytoplasm | kinase |  |
| 3.12E-05 | -2.523 | GGCT | gamma-glutamylcyclotransferase | cytoplasm | enzyme |  |
| 3.19E-05 | 2.101 | TBC1D4 | TBC1 domain family, member 4 | cytoplasm | ns |  |
| 3.41E-05 | 2.380 | BTBD11 | BTB (POZ) domain containing 11 | ns | transcription regulator |  |
| 3.44E-05 | -3.551 | PNP | purine nucleoside phosphorylase | nucleus | enzyme | forodesine, 9-deaza-9-(3-thienylmethyl)guanine |
| 3.66E-05 | -2.801 | PROS1 | protein S (alpha) | extracellular space | ns |  |
| 3.67E-05 | 2.463 | SLC16A2 | solute carrier family 16, member 2 (thyroid hormone transporter) | plasma membrane | transporter |  |
| 3.71E-05 | 2.502 | FRMD4B | FERM domain containing 4B | cytoplasm | ns |  |
| 3.81E-05 | 2.370 | GNAI1 | guanine nucleotide binding protein (G protein), alpha inhibiting activity polypeptide 1 | plasma membrane | enzyme |  |
| 3.97E-05 | 2.417 | KHDRBS2 | KH domain containing, RNA binding, signal transduction associated 2 | nucleus | ns |  |
| 4.05E-05 | -2.283 | ZNF714 | zinc finger protein 714 | ns | ns |  |
| 4.30E-05 | 2.431 | ANKRD18A | ankyrin repeat domain 18A | ns | ns |  |
| 4.59E-05 | 3.692 | ISM1 | isthmin 1, angiogenesis inhibitor | ns | ns |  |
| 4.73E-05 | 2.272 | NAIP | NLR family, apoptosis inhibitory protein | ns | ns |  |
| 4.81E-05 | 3.973 | LRP1B | low density lipoprotein receptor-related protein 1B | plasma membrane | transmembrane receptor |  |
| 4.96E-05 | -2.318 | B4GALNT3 | beta-1,4-N-acetyl-galactosaminyl transferase 3 | ns | enzyme |  |
| 5.12E-05 | 3.207 | ZMAT4 | zinc finger, matrin-type 4 | nucleus | ns |  |
| 5.20E-05 | -4.364 | KLK10 | kallikrein-related peptidase 10 | extracellular space | peptidase |  |
| 5.48E-05 | 2.210 | FAM155B | family with sequence similarity 155, member B | plasma membrane | transmembrane receptor |  |
| 5.56E-05 | -2.293 | MUC1 | mucin 1, cell surface associated | plasma membrane | transcription regulator | HuHMFG1 |
| 5.96E-05 | 2.119 | NEXN | nexilin (F actin binding protein) | plasma membrane | ns |  |
| 5.98E-05 | -2.170 | FXYD3 | FXYD domain containing ion transport regulator 3 | plasma membrane | ns |  |
| 6.03E-05 | -2.217 | PTPRE | protein tyrosine phosphatase, receptor type, E | plasma membrane | phosphatase |  |
| 6.32E-05 | 2.626 | PVRL3 | poliovirus receptor-related 3 | plasma membrane | ns |  |
| 6.59E-05 | -2.287 | DHRS3 | dehydrogenase/reductase (SDR family) member 3 | cytoplasm | enzyme |  |
| 7.34E-05 | 2.167 | ANGPT1 | angiopoietin 1 | extracellular space | growth factor | trebananib |
| 7.37E-05 | -2.544 | S100A14 | S100 calcium binding protein A14 | cytoplasm | ns |  |
| 7.43E-05 | -2.286 | ETV4 | ets variant 4 | nucleus | transcription regulator |  |
| 7.44E-05 | -2.974 | DSC3 | desmocollin 3 | plasma membrane | ns |  |
| 7.68E-05 | 2.392 | BOC | BOC cell adhesion associated, oncogene regulated | plasma membrane | ns |  |
| 8.64E-05 | -4.726 | GABRB2 | gamma-aminobutyric acid (GABA) A receptor, beta 2 | plasma membrane | ion channel | methohexital, aspirin/butalbital/ caffeine, aspirin/ butalbital and nss |
| 8.95E-05 | 2.955 | DNALI1 | dynein, axonemal, light intermediate chain 1 | cytoplasm | ns |  |
| 9.82E-05 | 3.531 | IQGAP2 | IQ motif containing GTPase activating protein 2 | cytoplasm | ns |  |
| 1.04E-04 | 3.316 | CWH43 | cell wall biogenesis 43 C-terminal homolog (S. cerevisiae) | ns | ns |  |
| 1.10E-04 | 2.163 | DLG2 | discs, large homolog 2 (Drosophila) | plasma membrane | kinase |  |
| 1.10E-04 | 2.269 | F3 | coagulation factor III (thromboplastin, tissue factor) | plasma membrane | transmembrane receptor | activated recombinant human factor VII |
| 1.13E-04 | 3.687 | TFCP2L1 | transcription factor CP2-like 1 | nucleus | transcription regulator |  |
| 1.15E-04 | -2.863 | IL1RN | interleukin 1 receptor antagonist | extracellular space | cytokine |  |
| 1.16E-04 | -2.336 | MACC1 | metastasis associated in colon cancer 1 | nucleus | ns |  |
| 1.18E-04 | 3.423 | TBX22 | T-box 22 | nucleus | transcription regulator |  |
| 1.22E-04 | 2.027 | CENPJ | centromere protein J | nucleus | transcription regulator |  |
| 1.46E-04 | -5.243 | MUC21 | mucin 21, cell surface associated | cytoplasm | ns |  |
| 1.64E-04 | 2.261 | ZBTB16 | zinc finger and BTB domain containing 16 | nucleus | transcription regulator |  |
| 1.71E-04 | -2.047 | ANXA9 | annexin A9 | plasma membrane | transmembrane receptor |  |
| 1.78E-04 | 3.386 | IPCEF1 | interaction protein for cytohesin exchange factors 1 | cytoplasm | enzyme |  |
| 1.89E-04 | 2.062 | CLCNKA | chloride channel, voltage-sensitive Ka | plasma membrane | ion channel |  |
| 1.94E-04 | 3.962 | NPR3 | natriuretic peptide receptor C/guanylate cyclase C (atrionatriuretic peptide receptor C) | plasma membrane | G-protein coupled receptor | nesiritide |
| 1.96E-04 | -2.176 | ELOVL7 | ELOVL fatty acid elongase 7 | cytoplasm | enzyme |  |
| 2.09E-04 | 2.158 | PHLDA1 | pleckstrin homology-like domain, family A, member 1 | cytoplasm | ns |  |
| 2.16E-04 | 2.091 | GABRB3 | gamma-aminobutyric acid (GABA) A receptor, beta 3 | plasma membrane | ion channel | methohexital, aspirin/ butalbital/ caffeine, and nss |
| 2.36E-04 | -3.627 | CRLF2 | cytokine receptor-like factor 2 | plasma membrane | transmembrane receptor |  |
| 2.48E-04 | -2.907 | SLPI | secretory leukocyte peptidase inhibitor | cytoplasm | ns |  |
| 2.54E-04 | -2.165 | RASD2 | RASD family, member 2 | cytoplasm | enzyme |  |
| 2.79E-04 | 2.723 | TMOD1 | tropomodulin 1 | cytoplasm | enzyme |  |
| 2.80E-04 | -2.130 | SHC3 | SHC (Src homology 2 domain containing) transforming protein 3 | cytoplasm | ns |  |
| 2.96E-04 | 2.028 | SUSD2 | sushi domain containing 2 | extracellular space | ns |  |
| 3.14E-04 | 3.207 | TNFRSF11B | tumor necrosis factor receptor superfamily, member 11b | plasma membrane | transmembrane receptor |  |
| 3.26E-04 | -2.842 | SPOCK2 | sparc/osteonectin, cwcv and kazal-like domains proteoglycan (testican) 2 | extracellular space | ns |  |
| 3.37E-04 | -3.580 | CITED1 | Cbp/p300-interacting transactivator, with Glu/Asp-rich carboxy-terminal domain, 1 | nucleus | transcription regulator |  |
| 3.42E-04 | -3.129 | LAMB3 | laminin, beta 3 | extracellular space | transporter |  |
| 3.55E-04 | -2.239 | KCNN4 | potassium intermediate/small conductance calcium-activated channel, subfamily N, member 4 | plasma membrane | ion channel | clotrimazole, ICA 17043 |
| 3.57E-04 | -2.298 | TNFRSF12A | tumor necrosis factor receptor superfamily, member 12A | plasma membrane | transmembrane receptor |  |
| 3.67E-04 | 2.376 | ENPP3 | ectonucleotide pyrophosphatase/phosphodiesterase 3 | plasma membrane | enzyme |  |
| 3.88E-04 | -2.476 | SFN | stratifin | cytoplasm | ns |  |
| 3.89E-04 | -2.617 | TRIM29 | tripartite motif containing 29 | cytoplasm | transcription regulator |  |
| 3.93E-04 | -2.664 | CRLF1 | cytokine receptor-like factor 1 | extracellular space | ns |  |
| 4.14E-04 | 3.080 | KIT | v-kit Hardy-Zuckerman 4 feline sarcoma viral oncogene homolog | plasma membrane | transmembrane receptor | dasatinib, sunitinib, pazopanib, tivozanib, motesanib, and nss |
| 4.22E-04 | 3.020 | MT1F | metallothionein 1F | ns | ns |  |
| 4.24E-04 | -2.154 | GALE | UDP-galactose-4-epimerase | cytoplasm | enzyme |  |
| 4.29E-04 | -2.359 | CDH4 | cadherin 4, type 1, R-cadherin (retinal) | plasma membrane | ns |  |
| 4.29E-04 | -2.086 | SNORA59B | small nucleolar RNA, H/ACA box 59B | ns | ns |  |
| 4.32E-04 | -2.222 | SDC4 | syndecan 4 | plasma membrane | ns |  |
| 4.34E-04 | -2.212 | FAM129A | family with sequence similarity 129, member A | cytoplasm | ns |  |
| 4.48E-04 | 2.897 | DIO2 | deiodinase, iodothyronine, type II | cytoplasm | enzyme |  |
| 4.53E-04 | -3.205 | CST6 | cystatin E/M | extracellular space | ns |  |
| 4.59E-04 | -2.075 | DUSP6 | dual specificity phosphatase 6 | cytoplasm | phosphatase |  |
| 4.66E-04 | -2.033 | GLRB | glycine receptor, beta | plasma membrane | ion channel |  |
| 4.73E-04 | 3.253 | CP | ceruloplasmin (ferroxidase) | extracellular space | enzyme |  |
| 4.80E-04 | 2.368 | PDE7B | phosphodiesterase 7B | cytoplasm | enzyme | dyphylline, nitroglycerin, aminophylline, anagrelide, and nss |
| 4.90E-04 | -2.598 | FN1 | fibronectin 1 | extracellular space | enzyme |  |
| 5.04E-04 | 6.831 | PKHD1L1 | polycystic kidney and hepatic disease 1 (autosomal recessive)-like 1 | extracellular space | ns |  |
| 5.11E-04 | 2.075 | TCF7L1 | transcription factor 7-like 1 (T-cell specific, HMG-box) | nucleus | transcription regulator |  |
| 5.30E-04 | 2.543 | FBN1 | fibrillin 1 | extracellular space | ns |  |
| 5.36E-04 | 3.976 | ZNF804B | zinc finger protein 804B | ns | ns |  |
| 5.47E-04 | 2.816 | KCNK2 | potassium channel, subfamily K, member 2 | plasma membrane | ion channel | dofetilide |
| 6.36E-04 | 3.525 | PLA2R1 | phospholipase A2 receptor 1, 180kDa | plasma membrane | transmembrane receptor |  |
| 6.54E-04 | 2.057 | FOXP2 | forkhead box P2 | nucleus | transcription regulator |  |
| 6.95E-04 | -2.137 | KCNQ3 | potassium voltage-gated channel, KQT-like subfamily, member 3 | plasma membrane | ion channel | ezogabine |
| 7.14E-04 | -2.822 | PDE5A | phosphodiesterase 5A, cGMP-specific | cytoplasm | enzyme | dyphylline, nitroglycerin, udenafil, and nss |
| 7.15E-04 | -3.033 | MMP16 | matrix metallopeptidase 16 (membrane-inserted) | extracellular space | peptidase | marimastat |
| 7.32E-04 | -2.261 | CTTNBP2 | cortactin binding protein 2 | cytoplasm | ns |  |
| 7.43E-04 | 3.300 | ST6GAL1 | ST6 beta-galactosamide alpha-2,6-sialyltranferase 1 | cytoplasm | enzyme |  |
| 7.53E-04 | -3.932 | SYTL5 | synaptotagmin-like 5 | ns | ns |  |
| 7.77E-04 | 2.611 | ANGPTL1 | angiopoietin-like 1 | plasma membrane | ns |  |
| 7.85E-04 | -2.111 | DUSP5 | dual specificity phosphatase 5 | nucleus | phosphatase |  |
| 7.99E-04 | 2.116 | CDH16 | cadherin 16, KSP-cadherin | plasma membrane | enzyme |  |
| 8.15E-04 | 2.465 | EDN3 | endothelin 3 | extracellular space | ns |  |
| 8.50E-04 | 2.592 | CD36 | CD36 molecule (thrombospondin receptor) | plasma membrane | transmembrane receptor |  |
| 8.51E-04 | -4.451 | CHI3L1 | chitinase 3-like 1 (cartilage glycoprotein-39) | extracellular space | enzyme |  |
| 8.65E-04 | 2.023 | MAPK4 | mitogen-activated protein kinase 4 | cytoplasm | kinase |  |
| 8.70E-04 | -2.609 | FRMD5 | FERM domain containing 5 | ns | ns |  |
| 8.70E-04 | 2.994 | MUM1L1 | melanoma associated antigen (mutated) 1-like 1 | ns | ns |  |
| 9.12E-04 | 2.018 | FCGBP | Fc fragment of IgG binding protein | extracellular space | ns |  |
| 9.23E-04 | -2.629 | CTSH | cathepsin H | cytoplasm | peptidase |  |
| 1.03E-03 | 2.100 | PCDHB15 | protocadherin beta 15 | plasma membrane | ns |  |
| 1.09E-03 | 2.055 | GPR155 | G protein-coupled receptor 155 | plasma membrane | G-protein coupled receptor |  |
| 1.10E-03 | 2.294 | PDE8B | phosphodiesterase 8B | cytoplasm | enzyme | ketotifen |
| 1.10E-03 | -3.321 | SYT1 | synaptotagmin I | cytoplasm | transporter |  |
| 1.11E-03 | 2.536 | PAK3 | p21 protein (Cdc42/Rac)-activated kinase 3 | cytoplasm | kinase |  |
| 1.16E-03 | -2.229 | FAM111B | family with sequence similarity 111, member B | ns | ns |  |
| 1.16E-03 | 2.883 | MT1G | metallothionein 1G | ns | ns |  |
| 1.18E-03 | -2.634 | ADCY8 | adenylate cyclase 8 (brain) | plasma membrane | enzyme |  |
| 1.22E-03 | 2.159 | LRRC7 | leucine rich repeat containing 7 | plasma membrane | ns |  |
| 1.24E-03 | 2.224 | ABCA8 | ATP-binding cassette, sub-family A (ABC1), member 8 | plasma membrane | transporter |  |
| 1.25E-03 | 3.812 | TFF3 | trefoil factor 3 (intestinal) | extracellular space | ns |  |
| 1.33E-03 | 2.687 | DGKI | diacylglycerol kinase, iota | cytoplasm | kinase |  |
| 1.33E-03 | 2.091 | PAPSS2 | 3'-phosphoadenosine 5'-phosphosulfate synthase 2 | cytoplasm | enzyme |  |
| 1.40E-03 | 2.261 | ALDH1A1 | aldehyde dehydrogenase 1 family, member A1 | cytoplasm | enzyme | disulfiram, chlorpropamide |
| 1.50E-03 | -2.019 | LPAR5 | lysophosphatidic acid receptor 5 | plasma membrane | G-protein coupled receptor |  |
| 1.50E-03 | -2.268 | MET | met proto-oncogene | plasma membrane | kinase | crizotinib, tivantinib, cabozantinib, INC280 |
| 1.50E-03 | -2.104 | VGLL1 | vestigial like 1 (Drosophila) | nucleus | transcription regulator |  |
| 1.59E-03 | -2.132 | S100A1 | S100 calcium binding protein A1 | cytoplasm | ns |  |
| 1.70E-03 | -2.065 | LY6E | lymphocyte antigen 6 complex, locus E | plasma membrane | ns |  |
| 1.73E-03 | 2.091 | RPS6KA6 | ribosomal protein S6 kinase, 90kDa, polypeptide 6 | cytoplasm | kinase |  |
| 1.79E-03 | -2.240 | SLC22A31 | solute carrier family 22, member 31 | ns | ns |  |
| 1.86E-03 | -2.298 | ALOX15B | arachidonate 15-lipoxygenase, type B | cytoplasm | enzyme |  |
| 1.88E-03 | 2.255 | MIR32 | micro RNA 32 | cytoplasm | micro RNA |  |
| 1.88E-03 | -2.185 | SLC30A2 | solute carrier family 30 (zinc transporter), member 2 | plasma membrane | transporter |  |
| 3.09E-05 | 2.224 | LOC643699 | golgin subfamily A member 8-like | ns | ns |  |
| 2.12E-04 | 2.121 | PROSP | protein S pseudogene (beta) | ns | ns |  |

**^1^fold change in BRAF^wt^ PTCs compared to BRAF^mut^ PTCs; ^2^ns, not specified**
