# Supplementary figures and images for: Effect of BRAF mutational status on expression profiles in conventional papillary thyroid carcinomas
Source: BMC Genomics. 2015 Jan 15;16(Suppl 1):S6. doi: 10.1186/1471-2164-16-S1-S6 (PMC4315163; doi:10.1186/1471-2164-16-S1-S6)

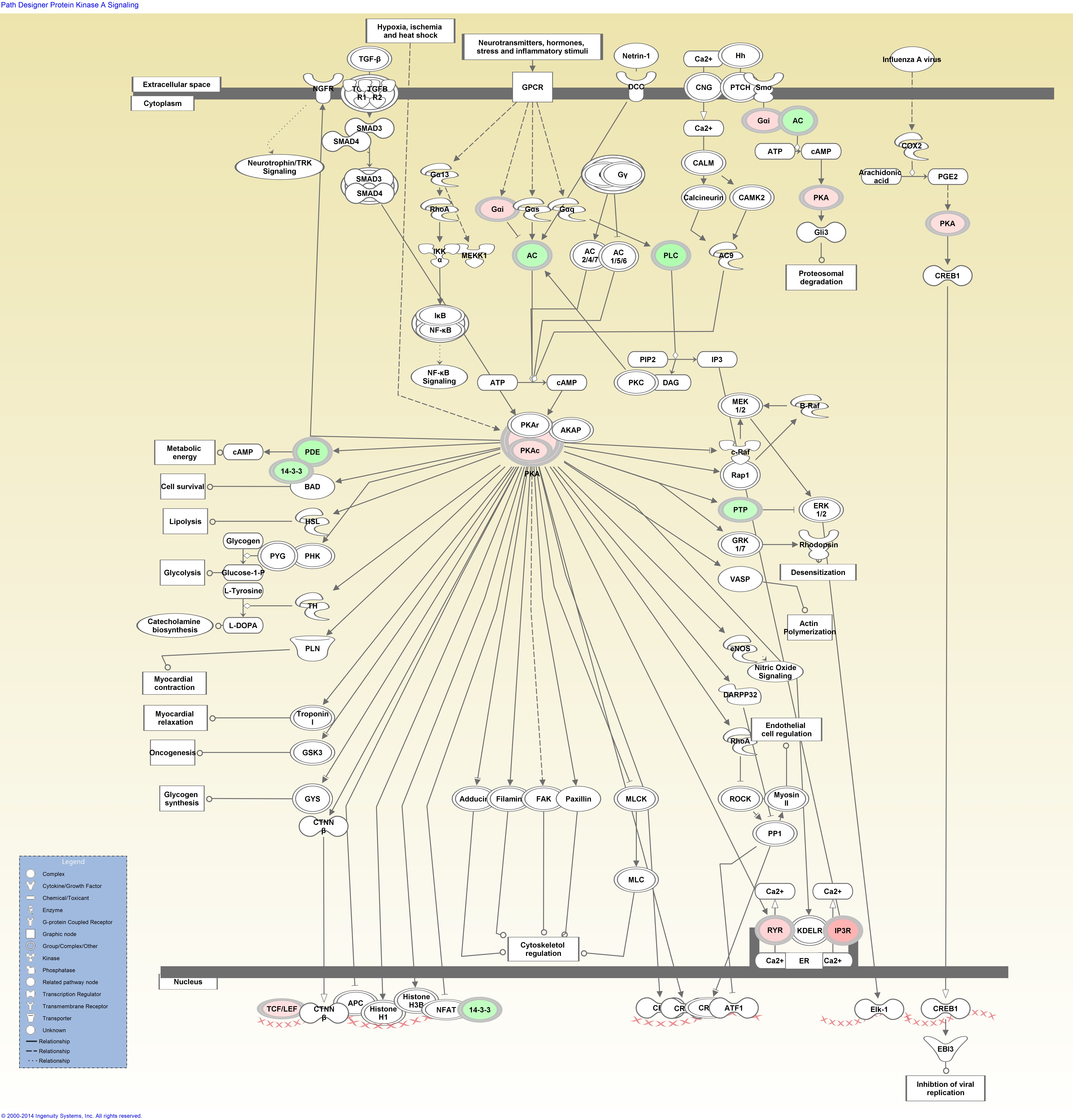

Supplement: Additional file 2 — The canonical PKA pathway is a second messenger cascade and involved in diverse functions as growth, development, metabolism, DNA replication/recombination, DNA repair and cellular organization. A number of molecules including members of PKA, ryanodine receptors (RYR), inositol trisphosphate receptors (IP3R), and lymphoid enhancing factors/T-cell factors (TCF/LEF) are upregulated (red) and number of molecules including members of phospholipases C (PLC), 14-3-3 proteins, and protein tyrosine phosphatases (PTP) are downregulated (green) in BRAFwt compared to BRAFmut PTCs. [file 1471-2164-16-S1-S6-S2.jpg]
